# The prognostic value of lymphocyte-albumin-neutrophil ratio variability in patients with breast cancer undergoing radiotherapy

**DOI:** 10.3389/fonc.2026.1892242

**Published:** 2026-08-03

**Authors:** Qianru Zhang, Zhe Wang, Jianing Jiang, Yiou Wang, Simiao Tian, Ruoyu Wang

**Affiliations:** 1 Department of Medical Oncology, Affiliated Zhongshan Hospital of Dalian University, Dalian, China; 2 Department of Medical Records and Statistics, Affiliated Zhongshan Hospital of Dalian University, Dalian, China

**Keywords:** breast cancer, dynamic monitoring, immune-nutritional status, lymphocyte-albumin-neutrophil Ratio (LANR), prognostic biomarker, radiotherapy

## Abstract

**Background:**

Traditional static biomarkers often fail to capture the ongoing immune-nutritional trajectory of cancer patients. This study aimed to evaluate the prognostic relevance of dynamic variation in the Lymphocyte-Albumin-Neutrophil Ratio (LANR) in patients with operable breast cancer (BC), with exploratory analyses according to postoperative radiotherapy (RT) status.

**Methods:**

We retrospectively evaluated women with BC with operable invasive breast cancer. LANR variability was quantified using the coefficient of variation (CV) of the pre-operative and post-chemotherapy measurements to represent the magnitude of relative variability between the two time points. Patients were categorized into high and low LANR variability groups using a data-derived cutoff. Overall survival (OS) and progression-free survival (PFS) were assessed using Kaplan-Meier curves and multivariable Cox proportional hazards models, including an exploratory interaction analysis according to postoperative radiotherapy (RT) status.

**Results:**

Among 1800 nonmetastatic BC women included in total, 8.2% (148/1800) death and 12% (216/1800) recurrence occurred after a median follow-up of 93 months (interquartile range, 73–116 months). In the overall cohort, the proportion of deaths was higher in the high-variability group (9.43% vs. 6.49%, P = 0.031), and the Kaplan–Meier comparison showed poorer OS (P = 0.032). In the fully adjusted model, the overall association was not statistically significant (HR = 1.34, 95% CI: 0.94–1.89, P = 0.11). The LANR variability-by-RT interaction was statistically significant for OS (P for interaction = 0.01) but not for PFS (P for interaction = 0.685). In the RT subgroup, high LANR variability was associated with poorer OS (HR = 1.82, 95% CI: 1.10–3.03, P = 0.02), whereas no clear association was observed in the non-RT subgroup (HR = 1.02, 95% CI: 0.63–1.66, P = 0.92).

**Conclusions:**

LANR variability is an inexpensive and readily available dynamic immune-nutritional indicator. A high LANR variability may help identify patients who require closer follow-up and monitoring, especially for those receiving postoperative radiotherapy. Further prospective validation and comparative analyses with established inflammatory and nutritional markers are required before clinical implementation.

## Introduction

Breast cancer remains the most frequently diagnosed malignancy and the leading cause of cancer-related mortality among women worldwide (1). The standard multidisciplinary management for operable breast cancer typically involves curative surgery followed by adjuvant therapies, including systemic chemotherapy, endocrine therapy, and locoregional radiotherapy (2). Currently, the American Joint Committee on Cancer (AJCC) TNM staging system serves as the cornerstone for risk stratification and therapeutic decision-making (3). However, breast cancer is a highly heterogeneous disease. Patients with identical TNM stages and seemingly similar clinicopathological features may still experience different survival outcomes. This clinical variability underscores the need for easily accessible, non-anatomic biological markers to refine individualized prognostic assessments and support postoperative monitoring.

Accumulating evidence highlights that tumor progression is not exclusively determined by the intrinsic genomic characteristics of the neoplastic cells, but is influenced by the host’s systemic inflammatory response and nutritional status (4). Peripheral blood leukocytes, particularly neutrophils, can promote tumor angiogenesis and metastasis by remodeling the tumor microenvironment (TME). Conversely, lymphocytes play a pivotal role in cytotoxic anti-tumor immunity, while serum albumin is a fundamental indicator of the host’s nutritional reserve and systemic inflammatory buffering capacity. Consequently, several blood-based indices—such as the neutrophil-to-lymphocyte ratio (NLR) and systemic immune-inflammation index (SII)—have been extensively explored, demonstrating significant prognostic value even in highly invasive subtypes such as triple-negative breast cancer (5). Recently, a novel composite index, the Lymphocyte-Albumin-Neutrophil Ratio (LANR), has emerged. By mathematically integrating inflammatory signals with nutritional status, baseline LANR has demonstrated robust prognostic value across diverse malignancies, including colorectal, gastric, and pancreatic cancers (6–9).

Despite these advancements, two major knowledge gaps hinder the clinical translation of these indices in breast cancer. First, traditional studies predominantly rely on static, single-timepoint baseline assessments prior to any intervention. However, tumor-host interactions are highly dynamic. Surgical trauma and cytotoxic chemotherapy may induce systemic immune remodeling and nutritional changes. Recent paradigms suggest that dynamic changes in systemic inflammatory markers over time may provide prognostic insights that static baseline values fail to capture (10). Second, the interplay between the host’s immuno-nutritional status and postoperative radiotherapy (RT) remains insufficiently explored, even though baseline systemic inflammation has been independently associated with late radiation-induced toxicities (11). While RT is crucial for locoregional control, it may expose circulating blood to radiation and contribute to radiation-induced lymphopenia (RIL) (12). Lymphocytes are radiosensitive, and RIL has been associated with poor long-term outcomes in several oncological settings (13). We hypothesize that marked LANR variability before RT may reflect an unfavorable immune-nutritional trajectory after surgery and chemotherapy, and that subsequent RT may act as an additional treatment-related immune stress in some patients. This hypothesis remains exploratory and requires direct validation using toxicity, lymphocyte trajectory, and treatment interruption data.

To address these critical gaps, we conducted a large-scale, retrospective cohort study of 1,800 patients with operable breast cancer. Diverging from conventional static evaluations, we aimed to dynamically quantify the LANR variability from the preoperative baseline to the post-chemotherapy assessment. Specifically, we sought to determine whether LANR variability was associated with long-term survival and to explore whether this prognostic association differed according to postoperative radiotherapy status.

## Materials and methods

### Study population

Data in the present study were obtained from the Affiliated Zhongshan Hospital of Dalian University. From January 2011 to December 2017, we retrospectively enrolled participants with invasive breast cancer who underwent curative primary tumor resection followed by a standard course of adjuvant chemotherapy. Participants were included in the current analysis if they met the following criteria: (1) pathologically confirmed invasive breast cancer (AJCC Stage I–III); (2) underwent curative surgery (mastectomy or breast-conserving surgery); (3) received at least 4 cycles of standard adjuvant chemotherapy; (4) age between 18 and 80 years; and (5) complete follow-up data available until the censoring date. Participants were excluded if they presented with distant metastasis (Stage IV) at diagnosis, concurrent active infections or hematological malignancies, a history of neoadjuvant therapy, or incomplete paired data regarding pre-operative and post-chemotherapy LANR parameters. Therefore, this led to a total enrollment of 1800 women with nonBC for the final analysis (flow-chart is shown in **Supplementary Figure 1**). The study was performed according to the guidelines of the Declaration of Helsinki and was approved by the Ethics Committee of the Affiliated Zhongshan Hospital of Dalian University (No. PJ-KY2026-088-1). All participants provided written informed consent (or consent was provided by their legal guardians) for the storage and analysis of their anonymized data before they were included in the study. The reporting of this study was guided by the Strengthening the Reporting of Observational Studies in Epidemiology (STROBE) statement, and the completed checklist will be provided as **Supplementary Material**.

### Clinical data collection

Clinical and clinicopathological data were retrospectively retrieved from the hospital’s electronic medical records system. Tumor-related characteristics were assessed based on the 8th edition of the American Joint Committee on Cancer (AJCC) TNM staging system, recording tumor size (T stage), lymph node involvement (N stage), histological subtype, and differentiation grade (3). Molecular subtypes, determined by the expression of estrogen receptor (ER), progesterone receptor (PR), human epidermal growth factor receptor 2 (HER2), and Ki-67 proliferation index, were also documented. Importantly, detailed information regarding postoperative radiotherapy status was systematically collected to support exploratory subgroup and interaction analyses.

### Measurement of dynamic LANR variability

To characterize the relative variability in LANR between the two measurements, peripheral blood samples were obtained at two time points: (1) baseline, within 1 week before primary tumor resection, and (2) after completion of adjuvant chemotherapy (within 1 week prior to the initiation of adjuvant radiotherapy). It is worth noting that all enrolled women with BC were inpatients, and these two measurements were obtained as part of routine clinical care rather than a study-specific sampling protocol. Hematological parameters, including white blood cell count, hemoglobin, absolute neutrophil count, absolute lymphocyte count, and serum albumin concentration, were measured by using a Sysmex XE-2100 automated hematology analyzer (Sysmex Corporation, Kobe, Japan) with a CV<5%, and retrieved from the laboratory information system. LANR was calculated as follows: LANR = (lymphocyte count × serum albumin)/neutrophil count. LANR variability was quantified using the coefficient of variation of the two LANR measurements, calculated as CV% = SD/mean × 100. This metric represents the magnitude of relative variability between the two time points and is direction-independent, because our primary hypothesis centers on systemic homeostatic stability rather than linear trajectory. Therefore, it does not distinguish an increase in LANR from a decrease in LANR, and we restricted the use of CV strictly in the text that it reflects instability, not clinical improvement or decline.

### Ascertainment of outcomes

Follow-up information was rigorously obtained through routine clinical surveillance, outpatient clinic visits, and telephone interviews. Patients were followed up at regular intervals after the completion of initial treatments. The primary endpoints were overall survival (OS) and progression-free survival (PFS). OS was defined as the time interval from the date of primary diagnosis to death from any cause. These endpoint definitions were applied consistently throughout all survival analyses, tables, and figures.

### Statistical analysis

Baseline characteristics were expressed as mean ± standard deviation (SD) or median with interquartile range according to distribution for continuous variables, and as frequency with percentage for categorical variables. Comparisons of characteristics between groups were performed using the Student’s t-test or Mann-Whitney U test for continuous variables, and the Chi-square test (χ²) or Fisher’s exact test for categorical variables. To determine the optimal prognostic cut-off value for this dynamic variability, receiver operating characteristic (ROC) was used with 1000 bootstrapped sample for validating the cutoff threshold. Based on this data-derived cutoff, patients were categorized into the high LANR variability group and the low LANR variability group. These categories represent greater versus lesser relative variability between the two measurements and do not indicate the direction of LANR change. The low LANR variability group was set as the reference category for all survival analyses. Survival curves were generated using the Kaplan-Meier method, and statistical differences between strata were assessed using the log-rank test. Multivariable Cox proportional hazards regression models were constructed to estimate the adjusted hazard ratios (HR) and 95% confidence intervals (CI), with adjustment for potential confounders including age, TNM stage, molecular subtype, immunotherapy, targeted therapy, endocrine therapy, nutritional status and radiotherapy. The proportional hazards assumption was assessed using Schoenfeld residual tests, which did not indicate a statistically significant violation of the assumption (global P = 0.06). To specifically elucidate the interaction between LANR variability and postoperative RT status, a two-way interaction term was constructed to test the interactive effect between LANR variability and radiotherapy for further subgroup analyses stratified by radiotherapy. In addition, the types of variability measure may influence the main findings; therefore, sensitivity analyses were conducted by using three other traditional metrics to differentiate the direction of LANR variation, and they were absolute, relative and log-ratio LANR change, which were calculated from post-chemotherapy/pre-radiotherapy to preoperative LANR. All statistical tests were two-sided, and a P-value < 0.05 was considered statistically significant. All analyses were performed using R software version 4.5.2 (14).

## Results

### Baseline clinicopathological characteristics

A total of 1,800 participants with breast cancer were included (mean age, 51.59 ± 9.74 years). Based on the data-derived cutoff, 740 patients were categorized as having low LANR variability and 1,060 as having high LANR variability, and the distribution of LANR variability was shown in **Supplementary Figure 2**.

Age, tumor size, TNM stage, and receptor status were similar between the two variability groups. Histological subtype (P = 0.008), serum albumin (P = 0.014), and total bilirubin (P = 0.033) differed between groups (**Table 1**).

**Table 1:** Baseline characteristics according to group of LANR variability (measured by coefficient of variation).

Variables	All sample (n=1800)	Low variability group (n=740)	High variability group (n=1060)	P value
TNM staging				0.361
I-II	1405 (78.06%)	586 (79.19%)	819 (77.26%)	
III	395 (21.94%)	154 (20.81%)	241 (22.74%)	
Histological subtype				0.008
Ductal Carcinoma	1620 (90.65%)	683 (93.18%)	937 (88.90%)	
Lobular Carcinoma	102 (5.71%)	29 (3.96%)	73 (6.93%)	
other	65 (3.64%)	21 (2.86%)	44 (4.17%)	
Differentiation grade				0.212
Well Differentiated	98 (6.19%)	47 (6.97%)	51 (5.62%)	
Moderately Differentiated	1283 (81.10%)	551 (81.75%)	732 (80.62%)	
Poorly Differentiated	201 (12.71%)	76 (11.28%)	125 (13.77%)	
CEA level				0.356
High	78 (4.96%)	37 (5.63%)	41 (4.48%)	
Low	1495 (95.04%)	620 (94.37%)	875 (95.52%)	
Ca-125 level				0.373
High	80 (4.78%)	38 (5.40%)	42 (4.33%)	
Low	1593 (95.22%)	666 (94.60%)	927 (95.67%)	
Ca 15–3 level				0.786
High	56 (3.36%)	22 (3.14%)	34 (3.51%)	
Low	1613 (96.64%)	678 (96.86%)	935 (96.49%)	
Molecular type				0.917
Luminal A	349 (19.39%)	139 (18.78%)	210 (19.81%)	
Luminal B	1016 (56.44%)	422 (57.03%)	594 (56.04%)	
HER2 positive	306 (17.00%)	128 (17.30%)	178 (16.79%)	
Triple negative	129 (7.17%)	51 (6.89%)	78 (7.36%)	
ER status				1
Negative	456 (25.33%)	187 (25.27%)	269 (25.38%)	
Positive	1344 (74.67%)	553 (74.73%)	791 (74.62%)	
PR status				0.606
Negative	612 (34.00%)	246 (33.24%)	366 (34.53%)	
Positive	1188 (66.00%)	494 (66.76%)	694 (65.47%)	
HER2 status				0.505
Negative	476 (26.47%)	189 (25.58%)	287 (27.10%)	
Positive	1322 (73.53%)	550 (74.42%)	772 (72.90%)	
Ki67 status				0.971
High	1335 (74.17%)	548 (74.05%)	787 (74.25%)	
Low	465 (25.83%)	192 (25.95%)	273 (25.75%)	
Age	51.59 (9.74)	51.44 (9.98)	51.70 (9.57)	0.58
Tumor size	2.57 (1.53)	2.58 (1.40)	2.57 (1.61)	0.845
Total Protein	74.51 (4.48)	74.54 (4.36)	74.49 (4.56)	0.789
Albumin	45.12 (2.77)	45.31 (2.63)	44.99 (2.86)	0.014
albumin-to-globulin ratio	1.57 (0.35)	1.58 (0.21)	1.57 (0.42)	0.647
Total Bilirubin	12.26 (3.34)	12.46 (3.29)	12.12 (3.36)	0.033
Alanine Aminotransferase	37.10 (17.09)	36.91 (16.05)	37.23 (17.78)	0.683
Aspartate Aminotransferase.	31.83 (10.68)	31.45 (9.57)	32.09 (11.39)	0.201
Urea	4.65 (1.13)	4.72 (1.30)	4.61 (0.99)	0.056
Creatinine	52.37 (7.25)	52.43 (6.94)	52.32 (7.46)	0.744
Uric acid	299.16 (56.87)	301.12 (55.51)	297.79 (57.79)	0.22
Cystatin C	0.89 (0.35)	0.87 (0.35)	0.90 (0.36)	0.09
White Blood Cell	5.96 (2.53)	5.35 (1.52)	6.39 (2.97)	<0.001
Neutrophil	4.26 (2.85)	3.49 (1.21)	4.81 (3.47)	<0.001
Lymphocytes	1.35 (0.43)	1.44 (0.38)	1.29 (0.46)	<0.001
Red Blood Cell	4.28 (3.40)	4.36 (3.76)	4.23 (3.13)	0.446
hemoglobin	124.06 (11.45)	125.61 (9.40)	122.97 (12.57)	<0.001
Platelets	251.44 (55.96)	251.86 (55.32)	251.14 (56.42)	0.788
Survival status				0.031
Alive	1652 (91.78%)	692 (93.51%)	960 (90.57%)	
Dead	148 (8.22%)	48 (6.49%)	100 (9.43%)	
PFS status				0.085
No	1547 (85.94%)	649 (87.70%)	898 (84.72%)	
Yes	253 (14.06%)	91 (12.30%)	162 (15.28%)	

Values are presented as mean (standard deviation), or n (%).

Ca 15-3, cancer antigen 15-3; CA-125, cancer antigen 125; CEA, carcinoembryonic antigen; ER, oestrogen receptor; PR, progesterone receptor; HER2, human epidermal growth factor receptor 2 amplification.PFS, progression-free survival.

At the post-chemotherapy assessment, the high-variability group had higher white blood cell and neutrophil counts (both P < 0.001) and lower lymphocyte counts, hemoglobin, and serum albumin than the low-variability group.

### Prognostic association of LANR index with survival outcomes

**Figure 1:**
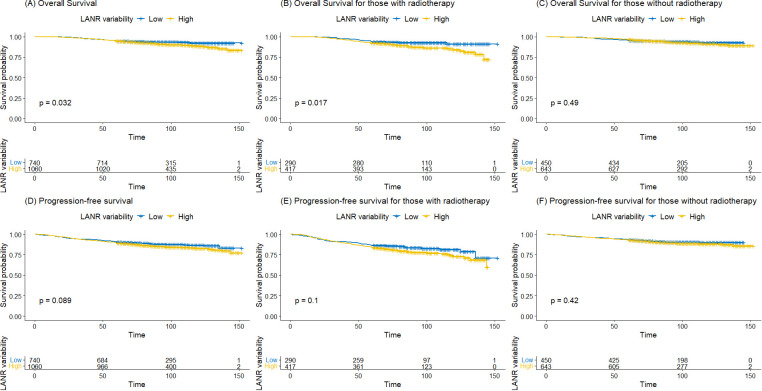
Kaplan-Meier (KM) curve analysis. Top panel: KM curve for overall survival in all sample **(A)** and sample with **(B)** and without **(C)** radiotherapy; bottom panel: KM curve for progression-free survival in all sample **(D)** and sample with **(E)** and without **(F)** radiotherapy.

During a median follow-up of 93 months (IQR, 73–116 months), 148 women with breast cancer had died and 216 events of recurrence occurred at the last follow-up. The proportion of deaths was higher in the high-variability group than in the low-variability group (9.43% vs. 6.49%, P = 0.031). KM analysis showed poorer OS in the high-variability group (log-rank P = 0.032, **Figure 1**). The PFS comparison did not reach statistical significance (log-rank P = 0.089).

In the age-adjusted Cox model (Model 1, **Table 2**), high LANR variability was associated with an HR for death of 1.41 (95% CI: 1.00–1.99, P = 0.05). After further adjustment in Model 3, the association in the overall cohort was not statistically significant (HR = 1.34, 95% CI: 0.94–1.89, P = 0.11).

**Table 2:** Association between groups of LANR variability (measured by coefficient of variation) and overall and progression-free survival.

Models	Low variability group	High variability group	P value
For overall survival			
Model 1	Reference	1.41 (1.00-1.99)	0.05
Model 2	Reference	1.33 (0.94-1.88)	0.107
Model 3	Reference	1.34 (0.94-1.89)	0.102
For progression-free survival			
Model 1	Reference	1.23 (0.95-1.59)	0.110
Model 2	Reference	1.18 (0.91-1.53)	0.204
Model 3	Reference	1.19 (0.92-1.54)	0.186

Model 1: adjusted for age;.

Model 2: further adjusted for TNM staging and molecular type based on ER, PR, HER2 and Ki-67.

Model 3: further adjusted for immunotherapy, targeted therapy, endocrine therapy, and radiotherapy.

Test for proportional HR: global 0.06.

The findings were replicated in sensitivity analysis when LANR variation was measured with absolute, relative, and log-ratio scale (**Supplementary Figure 3**). Women with decreased LANR had significantly poorer OS than their counterparts in the increased LANR group (log-rank P = 0.0018, **Supplementary Figure 3A**); however, only 17 women were classified into the decreased group. For the other two metrics, despite relatively balanced sample distributions, no significant differences in OS were observed (both log-rank P = 0.52; **Supplementary Figures 3B, C**).

### The prognostic value of dynamic LANR in patients undergoing radiotherapy

In the exploratory interaction analysis, the LANR variability-by-RT interaction was statistically significant for OS (P for interaction = 0.01) but not for PFS (P for interaction = 0.685). RT-stratified estimates were therefore interpreted as exploratory evidence of effect modification rather than proof that LANR variability predicts radiotherapy benefit.

**Figure 2:**
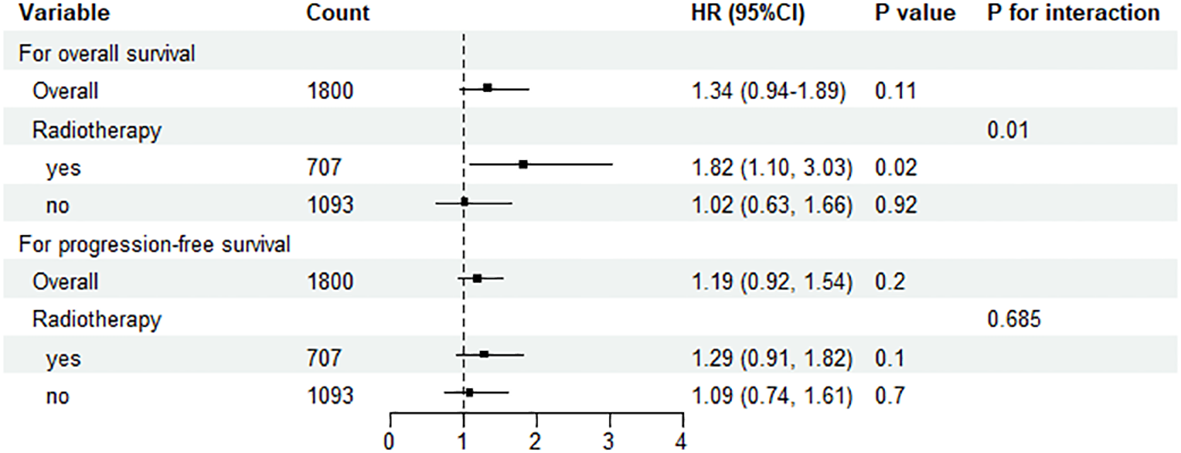
Hazard ratio of overall and progression-free survival by radiotherapy.

In the fully adjusted models shown in **Figure 2**, high LANR variability was associated with poorer OS in the radiotherapy subgroup (HR = 1.82, 95% CI: 1.10–3.03, P = 0.02), whereas no clear association was observed in the non-radiotherapy subgroup (HR = 1.02, 95% CI: 0.63–1.66, P = 0.92). For PFS, the estimates were HR = 1.29 (95% CI: 0.91–1.82, P = 0.10) in the RT subgroup and HR = 1.09 (95% CI: 0.74–1.61, P = 0.70) in the non-RT subgroup; the interaction was not statistically significant (P for interaction = 0.685).

## Discussion

In this retrospective cohort of 1,800 patients with invasive breast cancer, high LANR variability was associated with poorer OS in the postoperative RT subgroup after multivariable adjustment (HR = 1.89, 95% CI: 1.14–3.15, P = 0.01). In contrast, no clear association was observed in the non-RT subgroup (HR = 1.07, 95% CI: 0.66–1.73, P = 0.79). The LANR variability-by-RT interaction was statistically significant for OS but not for PFS. These findings should be regarded as exploratory evidence of possible effect modification rather than evidence that LANR variability predicts radiotherapy benefit. Furthermore, the sensitivity analysis showed differing results depending on how LANR variation was defined, indicating that the findings may be somewhat metric-dependent.

In the overall cohort, the Kaplan–Meier comparison showed poorer OS in patients with high LANR variability; however, the association did not reach statistical significance after full multivariable adjustment. The significant interaction observed for OS suggests that the prognostic association may differ according to postoperative RT status, although the subgroup findings should remain exploratory. Therefore, LANR variability should be interpreted as a potential prognostic marker in the postoperative RT population rather than as a validated predictive biomarker of radiotherapy benefit.

The association of systemic inflammation and nutritional status with cancer prognosis is well-established, yet the clinical utility of established biomarkers is often limited by their lack of sensitivity or comprehensive biological insight. Currently, the TNM staging system remains the gold standard for prognostication; however, it is inherently an anatomical classification that does not fully capture the biological heterogeneity of the host-tumor interface. The 8th edition of the AJCC Cancer Staging Manual explicitly encourages the integration of non-anatomic biologic markers to transition from a “population-based” to a “personalized” approach (3). In this context, blood-based immuno-nutritional indices offer a promising avenue for refining risk stratification.

Previous studies have utilized individual metrics, such as the neutrophil-to-lymphocyte ratio (NLR), platelet-to-lymphocyte ratio (PLR), or serum albumin alone, to predict outcomes in various malignancies. However, these markers reflect only a single dimension of the host’s status—either the inflammatory response or the nutritional reserve—and are susceptible to transient fluctuations caused by physiological stress or dehydration. The LANR index utilized in our study represents a potentially useful approach by integrating three critical components into a composite metric: neutrophils (drivers of tumor progression and metastasis), lymphocytes (effectors of anti-tumor immunity), and albumin (a surrogate for nutritional status and protein reserve). By mathematically combining these parameters, LANR provides a broader assessment of the “immuno-nutritional” landscape.

For instance, Wang et al. demonstrated in operable breast cancer that the LANR (calculated as L× A/N) had a higher area under the curve (AUC = 0.748) for predicting progression-free survival compared to albumin (AUC = 0.65) or neutrophils (AUC = 0.70) alone (15). Similarly, Liang et al. reported that in colorectal cancer, the composite LANR index was a more reliable independent prognostic factor than its individual components (7). Consistent with these findings, our data indicate that greater dynamic variation in this index (high LANR variability) may reflect an unstable host immune-nutritional status associated with poor long-term survival. This composite dynamic index may summarize concurrent changes in inflammatory, immune, and nutritional parameters within a single measure, potentially providing complementary information beyond anatomical TNM staging.

The Lymphocyte-Albumin-Neutrophil Ratio (LANR) is a relatively novel, multifaceted index that integrates the nutritional buffering capacity of serum albumin with the inflammatory and immunological signals of neutrophils and lymphocytes. Recently, the prognostic value of baseline LANR has been reported across a diverse spectrum of solid tumors. For instance, Liang et al. evaluated 753 patients with resectable colorectal cancer and identified a low preoperative LANR as an independent prognostic factor of both inferior overall survival (HR = 0.551) and disease progression (HR = 0.697) (7). Corroborating this evidence, a recent 2026 study by Xie et al. reported the clinical value of LANR in colorectal cohorts (9). Similarly, Zhuang et al. confirmed its prognostic value in resectable pancreatic ductal adenocarcinoma, noting that a low LANR independently correlates with adverse long-term survival (8). In gastric cancer, Li et al. demonstrated that nomograms incorporating LANR offer superior prognostic accuracy compared to traditional anatomical staging systems alone (6). Beyond the digestive system, the prognostic utility of LANR extends to non-gastrointestinal malignancies across different therapeutic modalities. In the surgical setting, Wang et al. evaluated a cohort of 202 patients with early-stage (stage IB-IIA) cervical cancer who underwent radical hysterectomy. They demonstrated that preoperative LANR exhibited prognostic accuracy, achieving an area under the curve (AUC) of 0.704 for predicting overall survival (OS) and 0.745 for progression-free survival (PFS) (16). Furthermore, extending its applicability to definitive radiotherapy, Zhang et al. investigated 805 patients with nasopharyngeal carcinoma. Their findings established that a lower pretreatment LANR independently predicted worse OS, PFS, and metastasis-free survival (MFS) in patients receiving definitive radiotherapy, facilitating the potential prognostic nomograms (17). Focusing on breast cancer, a recent analysis of 200 operable cases by Wang et al. confirmed that preoperative LANR provides potential prognostic accuracy (AUC = 0.748) for progression-free survival. This supports the potential role of LANR as a prognostic marker for long-term disease control (15). Furthermore, extending its applicability to definitive radiotherapy, in a recent retrospective observational study involving 114 breast cancer patients, Nafissi et al. evaluated the diagnostic capacity of various peripheral blood inflammatory indices. Utilizing ordinal logistic regression, they delineated the specific roles of these markers, demonstrating that while the neutrophil-to-lymphocyte ratio (NLR) primarily serves as a predictor for tumor grade (AUC = 0.652), LANR is significantly associated with distinct immunohistochemical profiles, most notably HER2 expression status (18). Collectively, these pan-cancer studies suggest that LANR may serve as a surrogate marker for host immune-nutritional vulnerability.

Conventional systemic inflammatory indices—such as the neutrophil-to-lymphocyte ratio (NLR), platelet-to-lymphocyte ratio (PLR), and systemic immune-inflammation index (SII)—have been widely investigated in breast cancer and other malignancies. NLR and PLR mainly reflect the balance between systemic inflammation and host immune status, whereas SII further incorporates platelet counts and may provide a more comprehensive assessment of systemic inflammatory activity (19–22). However, many previous studies have relied on static measurements at a single baseline time point, which may not fully capture the dynamic interaction among tumor progression, host immune response, and treatment-related systemic changes. Increasing evidence suggests that longitudinal changes in inflammatory indices may provide additional clinical information beyond baseline values alone. Dan et al. reported that, in breast cancer patients receiving neoadjuvant chemotherapy, dynamic change in NLR was independently associated with pathological complete response, whereas baseline NLR was not significant in multivariable analysis (20). Similarly, Kim et al. evaluated serial changes in NLR and PLR in 533 breast cancer patients and found that post-treatment inflammatory changes, rather than baseline NLR or PLR, were independently associated with delayed metastasis (21). Dynamic monitoring of NLR and PLR has also been reported to provide clinically relevant information in patients with advanced breast cancer receiving primary endocrine therapy (22). In the radiotherapy setting, radiotherapy-induced elevation of NLR has been associated with higher recurrence risk and poorer outcomes in breast cancer patients, further supporting the clinical relevance of treatment-related immune-inflammatory perturbation (23). Compared with ΔNLR and ΔPLR, evidence regarding dynamic SII in breast cancer remains relatively limited. Recent studies have begun to evaluate SII, prognostic nutritional index (PNI), and their variations after neoadjuvant chemotherapy, suggesting that ΔSII or combined ΔPNI–ΔSII scores may help predict treatment response (24). Nevertheless, available evidence for ΔSII is still mainly focused on neoadjuvant treatment efficacy rather than long-term survival outcomes, and its role in the post-chemotherapy/pre-radiotherapy interval remains insufficiently defined. Based on this dynamic biomarker framework, the present study introduced LANR variability to characterize changes in the lymphocyte-albumin-neutrophil ratio from the preoperative baseline to the post-chemotherapy assessment. Unlike ΔNLR, ΔPLR, or ΔSII, LANR variability incorporates serum albumin in addition to lymphocytes and neutrophils, thereby integrating inflammatory-immune status with nutritional reserve. This feature may be particularly relevant before radiotherapy, when patients have already experienced surgical stress and systemic chemotherapy. Recent breast cancer studies have also reported the prognostic relevance of LANR and albumin-related inflammatory/nutritional indices, supporting the potential value of combining immune-inflammatory and nutritional information in risk stratification (15, 25). Therefore, LANR variability may provide complementary immuno-nutritional information beyond conventional dynamic inflammatory indices. However, its incremental value over established markers such as ΔNLR, ΔPLR, and ΔSII should be interpreted cautiously and requires further validation through direct comparative analyses, such as C-index, net reclassification improvement, integrated discrimination improvement, or decision curve analysis.

The observed difference in the estimated associations between the radiotherapy subgroup (HR = 1.89) and the non-radiotherapy subgroup (HR = 1.07) may have several possible explanations (26). Among patients receiving postoperative RT, those with high LANR variability entered the radiotherapy phase with greater relative variability in immune-nutritional parameters. Radiotherapy, while highly effective for locoregional tumor control, may expose circulating blood to radiation, leading to radiation-induced lymphopenia (RIL). Lymphocytes are exquisitely radiosensitive. We hypothesize that for patients with high LANR variability before radiotherapy, the superimposition of regional radiotherapy may act as an additional treatment-related immune stress. This process may further impair anti-tumor immune surveillance and contribute to poorer long-term outcomes. Conversely, patients with lower LANR variability may have had a more preserved immune-nutritional profile during the peri-radiotherapy period. However, this mechanism remains hypothetical in the present study because serial lymphocyte counts during radiotherapy, radiotherapy-related toxicity, and treatment interruption data were not available. Therefore, high LANR variability should not be interpreted as direct evidence of poor radiotherapy tolerance, but rather as a potential marker for identifying patients who may require closer monitoring during postoperative treatment.

## Limitations

The present study has several limitations that should be mentioned. First, this was a single-center, retrospective observational study. Although we adjusted for multiple covariates, residual confounding by unmeasured factors and immortal time bias cannot be ruled out. For immortal time bias, the time interval between study entry and initiation of radiochemotherapy constitutes immortal time, since patients must remain event-free to receive subsequent adjuvant therapies. Second, we assessed LANR variability based on two specific time points (from the preoperative baseline to the post-chemotherapy assessment), biological and analytical variability could also attribute to the LANR variability, and this metric only reflects the magnitude of relative fluctuation and cannot capture the directional change of LANR. However, the sensitivity analysis by using other directional variation metrics between two time points did fit this study. In addition, the continuous dynamic fluctuation of LANR during and after the entire radiotherapy course was not evaluated and warrants future investigation to map the complete trajectory of immune recovery. Third, while our sample size was substantial (N = 1800), the radiotherapy-stratified findings require validation in external, multi-institutional cohorts to ensure generalizability. The rigorous and formal nutrition evaluation was not available due to retrospective nature, however, LANR is considered to integrate nutritional status in its definition. Finally, the specific biological mechanisms linking high LANR variability to poorer survival outcomes, such as local tumor hypoxia or specific T-cell subset depletion, were not directly measured in this clinical cohort.

## Conclusion

In this large retrospective cohort, high LANR variability was independently associated with inferior OS among patients receiving postoperative RT, and a significant LANR variability-by-RT interaction was observed for OS. These findings suggest that the prognostic relevance of LANR variability may differ according to postoperative RT status. This prognostic value is most pronounced in patients undergoing postoperative radiotherapy. The LANR variability represents a simple, cost-effective tool that could assist clinicians in identifying patients with poorer survival outcomes (indicated by a high LANR variability), thereby indicating a potential need for intensified supportive care or closer surveillance. For patients with high LANR variability before radiotherapy, closer clinical monitoring and supportive nutritional or immunological care may be considered. Further validation is required before LANR variability can be used to guide radiotherapy-related decision-making.

## Data Availability

The data analyzed in this study is subject to the following licenses/restrictions: The data in this study are available from the corresponding author upon reasonable request. Requests to access these datasets should be directed to tiansimiao@dlu.edu.cn.
