# Supplementary material for: The prognostic value of lymphocyte-albumin-neutrophil ratio variability in patients with breast cancer undergoing radiotherapy
Source: Front Oncol. 2026 Aug 3;16:1892242. doi: 10.3389/fonc.2026.1892242 (PMC13477883; doi:10.3389/fonc.2026.1892242)
Supplement: Supplementary file 1 [file DataSheet1.docx]

Supplementary Material

Supplementary Figure 1. Flow chart of patient selection for this cohort study.

3809 adult patients identified from hospital-based cohort, with a new histopathological diagnosis of breast cancer during the 2011-2017, were considered.

1800 adult patients aged 18-80 years with nonmetastatic breast cancer were included for this study samples

1936 adult patients aged 18-80 years with nonmetastatic breast cancer

1873 excluded

- Patients with aged $>$ 80 years (n=422)
- Patients with metastasis BC (n=455)
- Patients did not undergo surgery (n=108)
- unavailable vital status were (n=347)
- a history of neoadjuvant therapy (n=362)
- concurrent active infections or hematological malignancies (179)

Patients with incomplete paired LANR parameters were also excluded (n=136)

**Supplementary Figure 2.** Distribution of Lymphocyte-Albumin-Neutrophil Ratio (LANR) variability, measured by coefficient of variation.

**Supplementary Figure 3.** Kaplan-Meier (KM) curve of overall survival by (A) absolute, (B) log-ratio and (C) relative Lymphocyte-Albumin-Neutrophil Ratio (LANR) change.
